# Opportunistic and systematic screening for atrial fibrillation in adults aged ≥ 65 years in primary care: a systematic review of randomized and cluster randomized trials

**DOI:** 10.1186/s12872-026-06253-2

**Published:** 2026-09-11

**Authors:** Mohamed Ali Mansour, Mohamed Ezzat Ibrahim Hussein, Mohamed Shawky Al-Didamony Tantawy Foda

**Affiliations:** Faculty of Medicine, Zagazig University, Zagazig, Egypt

**Keywords:** Atrial fibrillation, Screening, Primary care, Opportunistic screening, Systematic screening

## Abstract

**Objectives:**

To evaluate whether opportunistic atrial fibrillation (AF) screening in adults aged ≥ 65 years improves AF detection and clinical outcomes compared with usual care, and to compare opportunistic and systematic screening strategies in terms of AF detection and clinical outcomes.

**Methods:**

A systematic review was conducted in accordance with PRISMA 2020 guidelines. Randomized and cluster-randomized controlled trials evaluating opportunistic or systematic AF screening in adults aged ≥ 65 years were included. Electronic databases (MEDLINE, Embase, and CENTRAL) were searched from inception to December 2024.

Studies with sufficiently comparable outcome data were included in a structured descriptive quantitative synthesis of AF detection rates. Because of substantial clinical and methodological heterogeneity among the included studies, a pooled meta-analysis was not performed. Instead, study-level outcomes were summarized descriptively. Studies with heterogeneous designs or outcome reporting were synthesized narratively. Outcomes of interest included AF detection, initiation of oral anticoagulation, stroke incidence, and all-cause mortality. Risk of bias was assessed using the Cochrane Risk of Bias 2.0 tool. Study selection and data extraction were performed independently by two reviewers.

**Results:**

Nine randomized or cluster-randomized trials were included. Both opportunistic and systematic screening approaches were associated with increased detection of previously undiagnosed AF compared with usual care.

Opportunistic screening increased AF detection in several trials; however, direct comparisons with systematic approaches should be interpreted cautiously because of heterogeneity in monitoring intensity, follow-up duration, and population characteristics.

Studies employing prolonged or continuous monitoring generally yielded higher AF detection rates; however, direct comparisons with opportunistic screening should be interpreted cautiously because of differences in monitoring intensity, follow-up duration, and population characteristics. Anticoagulation initiation varied across studies. Across studies, no consistent reduction in stroke incidence or all-cause mortality was observed. Most trials were judged to have some concerns regarding risk of bias, primarily related to pragmatic trial designs. Because AF diagnosis was confirmed using objective electrocardiographic criteria, the risk of outcome measurement bias was generally low.

**Conclusion:**

AF screening increases detection of previously undiagnosed AF in older adults. Opportunistic screening may be more readily integrated into routine healthcare encounters than organized systematic screening programs; however, implementation outcomes and cost-effectiveness were not consistently evaluated across the included trials. Current randomized evidence does not demonstrate consistent improvement in clinical outcomes such as stroke reduction or mortality, highlighting the need for further high-quality randomized trials evaluating integrated screening and post-detection care pathways.

**Supplementary Information:**

The online version contains supplementary material available at https://doi.org/10.1186/s12872-026-06253-2.

## Highlights

Opportunistic and systematic screening increase detection of atrial fibrillation in adults aged ≥ 65 years.

Randomized evidence supports improved detection but not consistent reduction in stroke or mortality.

Opportunistic screening may be more readily integrated into routine healthcare encounters than organized systematic screening programs; however, implementation outcomes were not consistently evaluated.

Targeted screening approaches may be appropriate for selected higher-risk populations.

**Supplementary Information:**

The online version contains supplementary material available at https://doi.org/10.1186/s12872-026-06253-2.

## Introduction

Atrial fibrillation (AF) is the most common sustained cardiac arrhythmia and a major contributor to ischemic stroke, heart failure, and mortality in older adults. Its prevalence increases substantially after the age of 65 years, and a considerable proportion of cases remain undiagnosed due to asymptomatic or intermittent presentation [1, 2].

Subclinical and intermittently detected AF has been increasingly recognized as a clinically relevant entity, contributing to thromboembolic risk, particularly in older adults with multimorbidity and underlying cardiovascular comorbidities [2, 3]. Advances in screening technologies, including handheld and wearable electrocardiographic devices, have enhanced the feasibility of detecting previously unrecognized AF outside conventional diagnostic settings [1, 2]. However, the clinical significance of screen-detected AF—especially short-duration or low-burden episodes—remains uncertain and continues to be an area of active investigation [2–4].

Early identification of AF enables timely initiation of oral anticoagulation, which has been shown to substantially reduce the risk of stroke [3]. Consequently, AF screening has emerged as a potential public health strategy. Two principal approaches have been evaluated: opportunistic screening, typically performed during routine primary care encounters, and systematic screening, which involves invitation-based programs or prolonged rhythm monitoring in defined populations.

In addition to detection yield, implementation factors such as healthcare resources, infrastructure, and monitoring requirements may influence the adoption of AF screening strategies in clinical practice. These considerations are relevant when interpreting the applicability of screening approaches across different healthcare settings.

Current international guidelines differ in their recommendations regarding AF screening [3, 4]. While several endorse opportunistic screening in individuals aged ≥ 65 years during routine healthcare encounters, population-wide systematic screening is not universally recommended. This variation reflects ongoing uncertainty regarding the balance between increased AF detection, downstream anticoagulation, potential harms, and health system costs.

Existing reviews have largely focused on AF detection yield without comprehensively integrating clinical outcomes, implementation considerations, and differences among screening strategies. Therefore, this systematic review aims to compare opportunistic and systematic AF screening strategies using evidence from randomized controlled trials, with an emphasis on clinically relevant outcomes and implementation considerations across heterogeneous screening strategies.

## Methods

### Study design and reporting

This systematic review was conducted in accordance with the Preferred Reporting Items for Systematic Reviews and Meta-Analyses (PRISMA) 2020 guidelines [5]. This review was conducted as a systematic review rather than a scoping review because it used predefined eligibility criteria, a structured search strategy, duplicate study selection, formal risk-of-bias assessment, and predefined outcome measures. The objective was to systematically evaluate and synthesize randomized evidence regarding AF screening effectiveness rather than to broadly map the available literature. A predefined protocol was developed prior to study selection. Although the protocol was not registered in PROSPERO, all methods were established a priori and adhered to PRISMA 2020 recommendations.

### Eligibility criteria

Studies were selected according to predefined inclusion and exclusion criteria based on the PICOS framework.

- Population: Adults aged ≥ 65 years in primary care or community-based settings.
- Intervention: Opportunistic or systematic screening for atrial fibrillation using pulse assessment or electrocardiographic methods.
- Comparator: Usual care or no screening.
- Outcomes: At least one of the following outcomes: AF detection, ischemic stroke, initiation of oral anticoagulation, or all-cause mortality.
- Study design: Randomized controlled trials or cluster-randomized trials.

Exclusion criteria included observational studies, studies without a comparator group, studies conducted exclusively in hospital settings, and studies not reporting relevant outcomes.

### Information sources and search strategy

A comprehensive literature search was conducted in MEDLINE (via PubMed), Embase, and the Cochrane Central Register of Controlled Trials (CENTRAL) from database inception to December 31, 2024. The final search was performed on January 5, 2025.

The search strategy combined Medical Subject Headings (MeSH) and free-text terms related to atrial fibrillation, screening, and primary care. No restrictions on publication date were applied. Only studies published in English were included.

Reference lists of included studies and relevant reviews were manually screened to identify additional eligible studies. Detailed database-specific search strategies for MEDLINE (PubMed), Embase, and CENTRAL are provided in Supplementary Material 1. The search strategies were adapted for each database using the appropriate controlled vocabulary and syntax.

### Study selection

All identified records were imported into reference management software, and duplicate records were removed.

Study selection was performed in two stages. First, two reviewers independently screened titles and abstracts to identify potentially eligible studies. Second, full-text articles of all potentially relevant studies were retrieved and assessed independently by two reviewers.

**Fig. 1:**
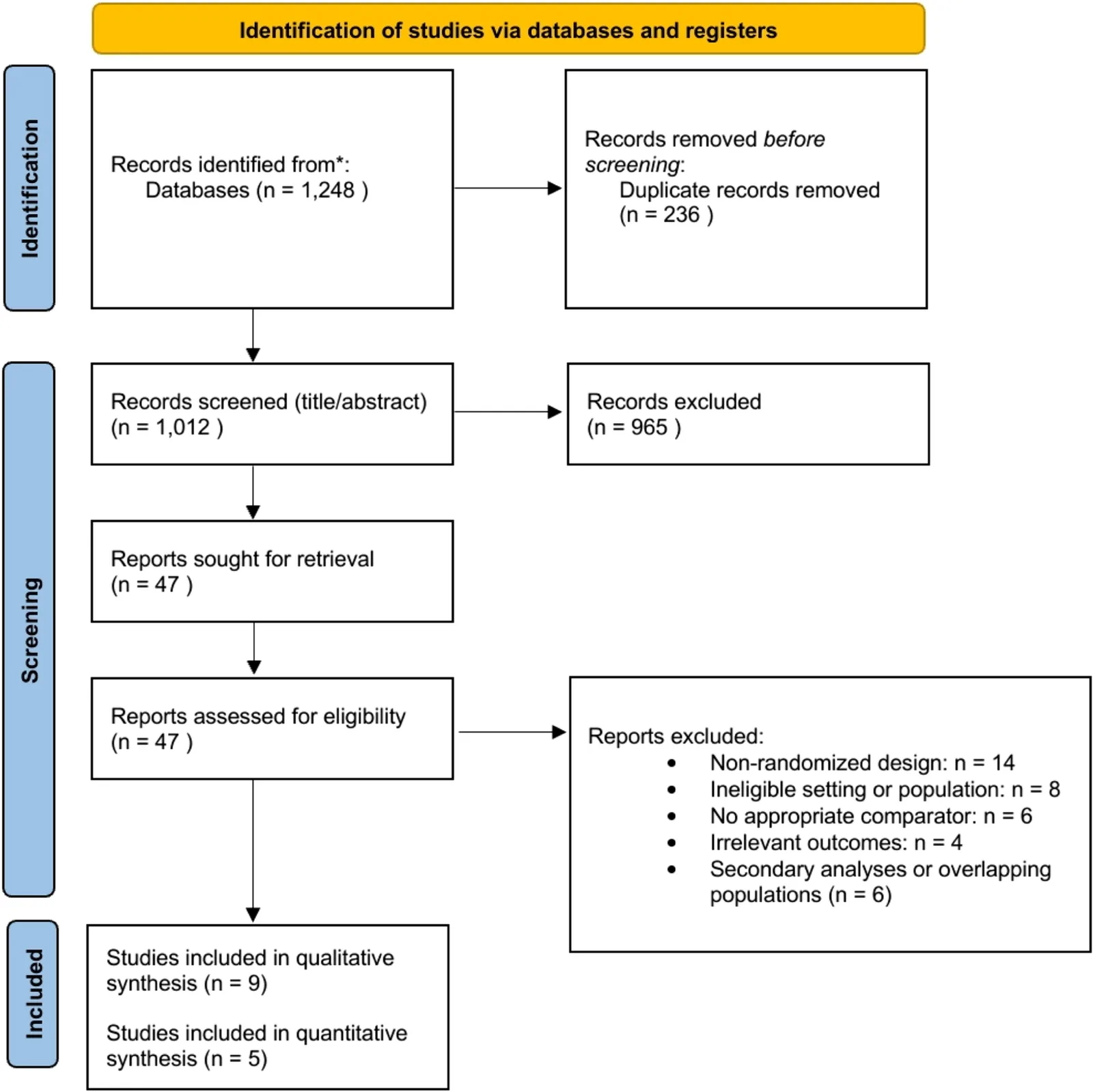
PRISMA 2020 flow diagram of study selection. Flow diagram illustrating the process of study identification, screening, eligibility assessment, and inclusion of randomized and cluster-randomized trials evaluating atrial fibrillation screening

Disagreements were resolved through discussion and consensus. Reasons for exclusion at the full-text stage were recorded and are presented in the PRISMA flow diagram (Fig. 1).

### Data collection process

Data were extracted independently by two reviewers using a standardized data extraction form. Discrepancies were resolved through discussion and consensus.

### Data items

The following data were extracted:

- Study characteristics (country, design, sample size).
- Population characteristics.
- Screening strategy (opportunistic or systematic).
- Duration of follow-up.
- AF detection rates.
- Initiation of oral anticoagulation.
- Stroke outcomes.
- Mortality outcomes.

When outcome definitions or study characteristics were incompletely reported, clarification was sought from supplementary publications when available. No numerical outcome data were imputed. Any interpretive assumptions regarding study characteristics were documented and are provided in Supplementary Material 2.

### Risk of bias assessment

Risk of bias was assessed independently by two reviewers using the Cochrane Risk of Bias 2.0 (RoB 2) tool across the following domains:

- Randomization process.
- Deviations from intended interventions.
- Missing outcome data.
- Measurement of outcomes.
- Selection of reported results.

Each study was classified as having low risk of bias, some concerns, or high risk of bias.

Formal assessment of reporting bias was not performed due to the limited number of studies and absence of meta-analysis.

### Effect measures

For AF detection, absolute differences in detection rates between screened and control groups were extracted or calculated. For clinical outcomes, reported effect estimates (e.g., risk ratios or hazard ratios) were extracted and summarized descriptively.

### Statistical analysis

Owing to substantial clinical and methodological heterogeneity among included studies, formal meta-analysis was not performed. AF detection outcomes were summarized descriptively using reported event rates and absolute differences between screened and control groups. Clinical outcomes were synthesized narratively.

### Synthesis methods

Due to heterogeneity in study design, screening strategies, outcome definitions, and reporting formats, a formal meta-analysis was not performed.

A structured synthesis approach was applied. Studies were included in the quantitative synthesis if they reported AF detection outcomes for both screened and control groups and provided sufficient numerical data to calculate absolute differences in detection rates. Studies with heterogeneous screening modalities, differing outcome definitions, varying monitoring durations, or insufficient numerical data were included in a narrative synthesis. Because of substantial heterogeneity in screening strategies, follow-up duration, and outcome reporting across studies, pooled meta-analysis was not considered appropriate.

This approach allowed transparent comparison of key outcomes while avoiding inappropriate statistical pooling of heterogeneous data.

## Results

### Study selection

The search identified 1,248 records, of which nine randomized or cluster-randomized trials met the eligibility criteria and were included in the review. Five trials reported sufficiently comparable AF detection data for inclusion in descriptive quantitative synthesis, while the remaining studies contributed to the narrative synthesis. The study selection process is presented in Fig. 1.

### Characteristics of included studies

Nine randomized or cluster-randomized trials were included [6–14], representing approximately 94,023 unique participants and evaluating a range of primary care, community-based, and device-based AF screening strategies. Two reports originated from the SAFE trial program and are presented separately because of differences in analysis and reporting; however, the Fitzmaurice et al. report represents a follow-up analysis of the SAFE cohort and was not considered an independent participant population.

Three large-scale trials (SAFE, STROKESTOP, and LOOP) contributed the majority of participants and outcome data [6, 8, 9]. The remaining studies were smaller trials conducted in primary care or community settings.

Screening approaches varied across studies and included:

- Opportunistic screening during routine clinical encounters using pulse palpation or single-time ECG.
- Systematic screening using repeated or scheduled ECG recordings.
- Device-based screening using handheld ECG devices or continuous monitoring systems.

For consistency throughout this review, screening approaches were categorized as opportunistic screening (screening during routine healthcare encounters), systematic screening (organized invitation-based screening programs), and device-based screening (screening using prolonged, continuous, wearable, or implantable monitoring technologies).

Follow-up durations ranged from 12 months to 6.9 years. Detailed study characteristics are presented in Table 1.

**Table 1:** Characteristics of included randomized and cluster-randomized trials

Study	Country	Design	Population	*N*	Screening Strategy	Comparator	Follow-up
SAFE (Hobbs et al., 2005) [6]	UK	Cluster RCT	≥ 65 years	14,802	Opportunistic/Systematic	Routine care	12 months
SAFE (Fitzmaurice et al., 2007) [7]	UK	Follow-up analysis	≥ 65 years	14,802	Opportunistic	Routine care	12 months
STROKESTOP (Svennberg et al., 2015) [8]	Sweden	RCT	≥ 75 years	28,768	Systematic intermittent ECG	Usual care	6.9 years
LOOP (Svendsen et al., 2021) [9]	Denmark	RCT	≥ 70 years	6,004	Implantable loop recorder	Standard care	5.4 years
REHEARSE-AF (Halcox et al., 2017) [10]	UK	RCT	≥ 65 years	1,001	Handheld ECG	Routine care	12 months
SCREEN-AF (Gladstone et al., 2021) [11]	Canada	RCT	≥ 65 years	856	Repeated ECG monitoring	Standard care	3 years
Uittenbogaart et al., 2020 [12]	Netherlands	Cluster RCT	≥ 65 years	9,218	Opportunistic screening	Usual care	12 months
VITAL-AF (Lubitz et al., 2022) [13]	USA	Cluster RCT	≥ 65 years	30,715	Point-of-care screening	Usual care	12 months
mSToPS (Steinhubl et al., 2018) [14]	USA	RCT	At-risk adults	2,659	Continuous ECG monitoring	Delayed monitoring	12 months

### Results of individual studies

Across the included trials, AF screening was associated with increased detection of previously undiagnosed atrial fibrillation compared with usual care. Detection rates varied according to the screening strategy used. Detailed findings from individual studies are summarized in Table 2.

**Table 2:** Effect of AF screening on detection and management outcomes

Study	Screening Approach	AF Detection Outcome	Anticoagulation Initiation	Clinical Outcome
SAFE (Hobbs et al., 2005) [6]	Opportunistic/Systematic Screening	Opportunistic screening: 75 new AF cases detected (1.64%/year) vs. 47 cases in controls (1.04%/year). Systematic screening: 74 new AF cases detected (1.62%/year).	Not reported	Stroke and mortality outcomes were not reported.
SAFE (Fitzmaurice et al., 2007) [7]	Opportunistic screening	Increased AF detection compared with routine care	Not reported	No clinical outcome data reported.
STROKESTOP (Svennberg et al., 2015) [8]	Systematic intermittent ECG screening	Previously undiagnosed AF detected in 3.0% of screened participants.	OAC initiated in approximately 3.7% of screened participants; >90% acceptance among newly diagnosed AF patients.	No significant reduction in mortality demonstrated.
LOOP (Svendsen et al., 2021) [9]	Implantable loop recorder	AF detected in 477/1501 (31.8%) screened participants vs. 550/4503 (12.2%) controls.	OAC initiated in 445/1501 (29.7%) vs. 591/4503 (13.1%).	Stroke/systemic embolism: 67/1501 (4.5%) vs. 251/4503 (5.6%); HR 0.80 (95% CI 0.61–1.05), not significant.
REHEARSE-AF (Halcox et al., 2017) [10]	Handheld ECG monitoring	AF detected in 19/500 (3.8%) screened participants vs. 5/501 (1.0%) controls.	Not reported	No significant difference in major clinical outcomes.
SCREEN-AF (Gladstone et al., 2021) [11]	Repeated ECG monitoring	AF detected in 31/434 (7.1%) screened participants vs. 5/422 (1.2%) controls.	OAC prescribed in 18/434 (4.1%) vs. 4/422 (0.9%)	No significant reduction in stroke, hospitalization, or mortality.
Uittenbogaart et al., 2020 [12]	Opportunistic screening	Increased AF detection compared with usual care.	Not reported	No significant difference in clinical outcomes reported.
VITAL-AF (Lubitz et al., 2022) [13]	Point-of-care screening	No significant increase in overall AF detection compared with usual care.	OAC initiated in 73.5% of newly diagnosed AF patients vs. 70.8% in controls.	No significant reduction in stroke or mortality.
mSToPS (Steinhubl et al., 2018) [14]	Continuous ECG monitoring	Higher AF detection compared with routine care.	Anticoagulant initiation rate: 5.7 vs. 3.7 per 100 person-years	Long-term clinical outcome benefit remained uncertain

### Quantitative synthesis

Five trials reported sufficiently comparable AF detection outcomes to permit a structured descriptive quantitative synthesis [6–9, 11]. Because of substantial clinical and methodological heterogeneity among the included studies, a pooled meta-analysis was not performed. Instead, study-level AF detection outcomes were summarized descriptively. Screening was consistently associated with increased detection of previously undiagnosed atrial fibrillation compared with usual care across the included studies. The descriptive quantitative findings are summarized in Table 3.

**Table 3:** Study-Level AF detection results included in the descriptive quantitative synthesis

Study	AF Detection (Screened Group)	AF Detection (Control Group)	Absolute Difference
SAFE (Hobbs et al., 2005) [6]	(1.64%) 75/4575	(1.04%) 47/4513	+ 0.60%
SAFE (Fitzmaurice et al., 2007) [7]	(1.62%) 74/4562	(1.04%) 47/4513	+ 0.58%
STROKESTOP (Svennberg et al., 2015) [8]	(3.0%) 218/7173	(0.9%) in control population	+ 2.1%
LOOP (Svendsen et al., 2021) [9]	(31.8%) 477/1501	(12.2%) 550/4503	+ 19.6%
SCREEN-AF (Gladstone et al., 2021) [11]	(7.1%) 31/434	(1.2%) 5/422	+ 5.9%

The Fitzmaurice et al. (2007) [7] publication represents a follow-up analysis of the SAFE cohort reported by Hobbs et al. (2005) [6]. Although presented separately because it reports different analyses, it was not considered an independent participant population and was not counted separately in the total number of participants included in this review

### Atrial fibrillation detection

Across the studies included in the quantitative synthesis, screening was associated with increased detection of previously undiagnosed atrial fibrillation compared with usual care. Absolute differences in AF detection ranged from 0.58% to 19.6% across included trials. Detailed AF detection outcomes are summarized in Tables 2 and 3.

### Anticoagulation initiation

Oral anticoagulation initiation was reported in several studies and generally occurred more frequently in screened participants than in control groups. In the LOOP trial, oral anticoagulation was initiated in 445/1501 (29.7%) screened participants compared with 591/4503 (13.1%) controls [9]. In SCREEN-AF, oral anticoagulation was prescribed in 18/434 (4.1%) screened participants compared with 4/422 (0.9%) controls [11]. In STROKESTOP, oral anticoagulation was initiated in approximately 3.7% of screened participants, with more than 90% acceptance among newly diagnosed AF patients [8]. Detailed anticoagulation outcomes are summarized in Table 2.

### Clinical outcomes

Clinical outcomes were reported inconsistently across the included trials. No statistically significant reduction in ischemic stroke or all-cause mortality was demonstrated in the studies that evaluated these outcomes [8, 9, 11]. Detailed outcome data are summarized in Table 4.

**Table 4:** Impact of screening on major clinical outcomes

Outcome	Overall Finding from Included Trials
AF detection	Screening consistently increased detection of previously undiagnosed atrial fibrillation compared with usual care.
Anticoagulation use	Increased anticoagulation initiation was reported in several studies following AF detection, although uptake varied across trials.
Stroke reduction	No trial demonstrated a statistically significant and consistent reduction in stroke incidence attributable to screening.
Mortality	No consistent mortality benefit was demonstrated across included studies.

### Studies not included in quantitative synthesis

Four studies were not included in the quantitative synthesis due to heterogeneity in outcome reporting, study design, or insufficient availability of comparable data.

These studies contributed to the qualitative synthesis, providing additional context on screening strategies, implementation feasibility, and variation in clinical outcomes.

### Risk of bias

Overall, most included trials were judged to have some concerns regarding risk of bias. Because AF diagnosis was confirmed using objective electrocardiographic criteria, lack of participant blinding was unlikely to introduce substantial detection bias. However, awareness of screening assignment may have influenced healthcare utilization, diagnostic evaluation, treatment decisions, and follow-up practices. Detailed risk-of-bias assessments are presented in Table 5.

**Table 5:** Risk of bias assessment (Cochrane RoB 2 Tool)

Study	Randomization process	Deviations from intended intervention	Missing outcome data	Outcome measurement	Selective reporting	Overall risk
SAFE (Hobbs et al., 2005) [6]	Low risk	Some concerns	Low risk	Low risk	Low risk	Some concerns
SAFE (Fitzmaurice et al., 2007) [7]	Some concerns	High	Low risk	Low risk	Low risk	High
STROKESTOP (Svennberg et al., 2015) [8]	Low risk	Some concerns	Low risk	Low risk	Low risk	Some concerns
LOOP (Svendsen et al., 2021) [9]	Low risk	Some concerns	Low risk	Low risk	Low risk	Some concerns
REHEARSE-AF (Halcox et al., 2017) [10]	Some concerns	Some concerns	Low risk	Low risk	Low risk	Some concerns
SCREEN-AF (Gladstone et al., 2021) [11]	Low risk	Some concerns	Low risk	Low risk	Low risk	Some concerns
Uittenbogaart et al., 2020 [12]	Low risk	Some concerns	Low risk	Low risk	Low risk	Some concerns
VITAL-AF (Lubitz et al., 2022) [13]	Low risk	Some concerns	Low risk	Low risk	Low risk	Some concerns
mSToPS (Steinhubl et al., 2018) [14]	Some concerns	Some concerns	Low risk	Low risk	Low risk	Some concerns

Most included trials were judged to have some concerns regarding overall risk of bias, while one study was judged to be at high risk of bias because one RoB 2 domain was rated high. The main concerns reflected the pragmatic nature of screening interventions and the inability to blind participants or healthcare providers. Because atrial fibrillation diagnosis was confirmed using objective electrocardiographic criteria, the risk of outcome measurement bias was generally low across studies

## Discussion

### Principal findings

This systematic review of randomized and cluster-randomized trials demonstrates that screening for atrial fibrillation (AF) in adults aged ≥ 65 years increases the detection of previously undiagnosed AF compared with usual care. Both opportunistic and systematic screening strategies were associated with improved detection across diverse healthcare settings. Importantly, not all included studies were conducted in routine primary-care settings. Several trials were population-based or employed device-intensive monitoring strategies, which may limit the generalizability of findings to routine primary-care practice.

However, despite increased detection and modest increases in anticoagulation initiation in some studies, no consistent reduction in ischemic stroke or all-cause mortality was observed.

The observed increase in AF detection across screening strategies reflects the ability of both opportunistic and systematic approaches to identify previously unrecognized AF. Studies employing repeated or continuous monitoring generally reported higher AF detection rates; however, direct comparisons across screening strategies should be interpreted cautiously because of substantial differences in study design, monitoring intensity, follow-up duration, participant characteristics, and screening settings. Although opportunistic screening may be incorporated into routine healthcare encounters, implementation outcomes, scalability, and cost-effectiveness were not consistently evaluated across the included randomized trials. The clinical relevance of detecting short-duration or subclinical AF also remains uncertain.

Importantly, increased detection did not consistently translate into improved clinical outcomes. Several explanations may account for the absence of consistent reductions in stroke or mortality despite increased AF detection. These include limited statistical power, detection of lower-burden or subclinical AF, variability in anticoagulation uptake, delays in treatment initiation, and competing risks in older populations. Similar findings have been reported in previous AF screening studies and systematic reviews, which have consistently demonstrated improved AF detection but uncertain effects on major clinical outcomes such as stroke and mortality [1, 2].

An important limitation of the available evidence is that several included trials were not designed or powered to detect differences in stroke or mortality outcomes. Consequently, the absence of demonstrated benefit for these endpoints should be interpreted cautiously, as failure to detect a statistically significant effect does not necessarily indicate absence of clinical benefit.

Recent real-world registry data complement the randomized screening literature by emphasizing that the clinical value of AF detection depends on subsequent management quality. In the Heidelberg HERA-FIB registry, inappropriate direct oral anticoagulant dosing was common among patients with AF treated with direct oral anticoagulants, and under-dosing was associated with a higher risk of adverse composite outcomes compared with appropriate dosing. These findings suggest that screening programs may benefit from including structured anticoagulation optimization rather than detection alone [15].

Real-world analyses of very elderly patients with AF also highlight that adults aged ≥ 80 years represent a high-risk subgroup in whom anticoagulation decisions, bleeding risk, comorbidity, and follow-up require careful individualized assessment. Patients without oral anticoagulation had worse clinical outcomes, while direct oral anticoagulant use increased over time, supporting the need for structured post-screening treatment optimization following AF detection, particularly in adults aged ≥ 80 years [16].

In addition, biomarker-enhanced risk stratification, including high-sensitivity cardiac troponin T added to CHA₂DS₂-VASc, may help refine post-detection risk assessment, although prospective validation is required before implementation [17]. Together, these data suggest that future screening pathways may benefit from evaluation as integrated care models that include ECG confirmation, risk stratification, anticoagulation initiation, dose verification, and follow-up.

AF screening should therefore not be evaluated as a standalone diagnostic intervention, but as part of a care pathway including ECG confirmation, CHA₂DS₂-VASc and bleeding-risk assessment, anticoagulation initiation, dose verification, and follow-up [2–4, 15–17].

Emerging evidence also suggests that clinical complexity may influence the potential benefits of AF screening. Older adults with AF frequently present with multimorbidity, frailty, polypharmacy, and competing risks that affect thromboembolic risk, bleeding risk, treatment decisions, and long-term outcomes. Recent analyses have demonstrated substantial heterogeneity in risk profiles among clinically complex AF populations across different healthcare settings and geographic regions [18]. This heterogeneity may partly explain why increased AF detection does not consistently translate into reductions in stroke or mortality across screening trials. Future studies should evaluate AF screening within the broader context of clinical complexity and individualized management strategies.

A comparison of opportunistic and systematic screening strategies across key domains is provided in Table 6.

**Table 6:** Pragmatic comparison of screening approaches based on included trials

Domain	Opportunistic Screening	Systematic Screening
Implementation Considerations	Conducted during routine primary-care encounters in included studies	Requires organized screening programs and supporting infrastructure
Cost/resource considerations	Resource utilization was not consistently evaluated across included studies	Often involved organized screening programs or monitoring protocols
Scalability	Scalability was not directly evaluated in included trials	Dependent on healthcare resources and infrastructure
Detection yield	Increased AF detection observed across included trials	Increased AF detection observed across included trials, including studies using repeated or prolonged monitoring
Health system applicability	Applicability within routine primary-care settings was described in several included studies.	Context-dependent and influenced by system capacity and resources
Resource requirements	Resource requirements were not consistently evaluated across included trials	Greater resource requirements, including personnel, equipment, and follow-up systems

These observations represent an interpretive synthesis of trial characteristics and implementation requirements derived from the included studies and should not be interpreted as comparative effect estimates

### Comparison with previous literature

Previous systematic reviews have reported increased AF detection associated with screening strategies, often emphasizing technological advances such as wearable and implantable devices [1, 2]. By restricting inclusion to randomized evidence and directly comparing opportunistic and systematic approaches, the present review provides a more clinically focused synthesis.

The findings are consistent with prior literature indicating that while screening improves detection, its impact on clinically meaningful outcomes remains uncertain [1, 2]. These results reinforce the distinction between diagnostic effectiveness and clinical effectiveness.

### Clinical and public health implications

From a clinical perspective, opportunistic AF screening can be incorporated into routine healthcare encounters. However, implementation outcomes, resource utilization, scalability, and cost-effectiveness were not consistently evaluated across the included randomized trials.

Despite increased detection of previously undiagnosed AF, the absence of consistent evidence for stroke reduction suggests that screening alone may be insufficient to improve clinical outcomes. Effective screening programs likely require integration with structured care pathways, including confirmatory diagnosis, risk stratification, and timely initiation of anticoagulation [2–4].

Systematic screening strategies may be appropriate for selected higher-risk populations. Studies employing organized or prolonged monitoring generally reported higher AF detection rates; however, direct comparisons with opportunistic screening should be interpreted cautiously because of differences in study design, monitoring intensity, follow-up duration, and participant characteristics [3, 4]. Their impact on clinically important outcomes remains uncertain, and implementation decisions should consider available healthcare resources, infrastructure, and local practice settings.

### Limitations

This review has several limitations. First, heterogeneity in screening strategies, populations, and outcome reporting limited the feasibility of meta-analysis. Second, pragmatic trial designs introduced potential performance bias because blinding of participants and healthcare providers was generally not feasible. Because atrial fibrillation diagnosis was confirmed using objective electrocardiographic criteria, lack of participant blinding was unlikely to introduce substantial detection bias. However, awareness of screening assignment may have influenced healthcare utilization, diagnostic evaluation, treatment decisions, and follow-up practices.

Additionally, the review protocol was not prospectively registered in PROSPERO or another systematic review registry. The absence of prospective registration may increase the risk of selective reporting and reduce transparency regarding protocol development, although predefined methods were established and followed throughout the review process. The structured synthesis approach was adopted to ensure appropriate interpretation of heterogeneous data.

## Conclusion

Opportunistic and systematic screening for atrial fibrillation in older adults increases the detection of previously undiagnosed AF. Current randomized evidence does not demonstrate consistent improvement in clinical outcomes such as stroke reduction or mortality, and implementation outcomes and cost-effectiveness were not consistently evaluated across the included trials. Future adequately powered randomized studies should determine whether integrating AF screening with optimized clinical management pathways can translate increased detection into meaningful improvements in patient outcomes.

## Supplementary Information

Supplementary Material 1.

Supplementary Material 2.

## Data Availability

All data extracted and analyzed during this study are included within the article.
