# Supplementary material for: Opportunistic and systematic screening for atrial fibrillation in adults aged ≥ 65 years in primary care: a systematic review of randomized and cluster randomized trials
Source: BMC Cardiovasc Disord. 2026 Sep 11;26:805. doi: 10.1186/s12872-026-06253-2 (PMC13584378; doi:10.1186/s12872-026-06253-2)
Supplement: Supplementary file 1 [file 12872_2026_6253_MOESM1_ESM.docx]

**Detailed Search Strategies**

**Search Date**

The electronic literature search was conducted from database inception through December 31, 2024. The final search was performed on January 5, 2025.

**Databases Searched**

1. MEDLINE (via PubMed)
2. Embase
3. Cochrane Central Register of Controlled Trials (CENTRAL)

**Eligibility Restrictions**

- Human studies
- English language publications
- Randomized controlled trials or cluster-randomized trials
- Adults aged ≥65 years
- Primary care or community-based screening settings

**MEDLINE (PubMed)**

("atrial fibrillation"[MeSH Terms] OR

"atrial fibrillation"[Title/Abstract] OR

AF[Title/Abstract])

AND

("screening"[MeSH Terms] OR

screening[Title/Abstract] OR

"early detection"[Title/Abstract])

AND

("primary care"[Title/Abstract] OR

"general practice"[Title/Abstract])

AND

(randomized controlled trial[Publication Type] OR

randomized[Title/Abstract] OR

randomly[Title/Abstract])

**Embase**

The PubMed strategy was adapted using Emtree terminology and Embase syntax.

('atrial fibrillation'/exp OR

'atrial fibrillation':ti,ab OR

AF:ti,ab)

AND

('screening'/exp OR

screening:ti,ab OR

'early detection':ti,ab)

AND

('primary care':ti,ab OR

'general practice':ti,ab)

AND

('randomized controlled trial'/exp OR

randomized:ti,ab OR

randomly:ti,ab)

Filters applied:

- Humans
- English language

**Cochrane Central Register of Controlled Trials (CENTRAL)**

("atrial fibrillation" OR AF)

AND

(screening OR "early detection")

AND

("primary care" OR "general practice")

AND

(randomized OR randomly OR trial)

No additional methodological filters were applied because CENTRAL primarily indexes controlled trials.

**Additional Search Methods**

Reference lists of all included studies and relevant review articles were manually screened to identify additional eligible studies not captured through database searches.

No unpublished studies, conference abstracts, dissertations, or gray literature sources were included.
