# Supplementary material for: Opportunistic and systematic screening for atrial fibrillation in adults aged ≥ 65 years in primary care: a systematic review of randomized and cluster randomized trials
Source: BMC Cardiovasc Disord. 2026 Sep 11;26:805. doi: 10.1186/s12872-026-06253-2 (PMC13584378; doi:10.1186/s12872-026-06253-2)
Supplement: Supplementary file 2 [file 12872_2026_6253_MOESM2_ESM.docx]

**Documentation of Interpretive Assumptions Used During Data Extraction**

**Purpose**

This supplementary file documents all interpretive assumptions made during study selection and data extraction.

No numerical outcome data were imputed, estimated, or mathematically derived beyond values explicitly reported in the original publications.

Where reporting was incomplete, assumptions were limited to descriptive study characteristics and screening strategy classification.

**Assumption 1: Classification of Screening Strategy**

**Studies affected**

- SAFE (Hobbs et al., 2005)
- SAFE Follow-up Analysis (Fitzmaurice et al., 2007)

**Issue**

The SAFE trial evaluated both opportunistic and systematic screening approaches.

**Assumption Applied**

The study was classified according to the specific screening arm being evaluated in the corresponding publication.

**Rationale**

This classification reflected the intervention reported by the original authors and enabled consistent comparison across included trials.

**Assumption 2: Classification of Device-Based Screening**

**Studies affected**

- LOOP (Svendsen et al., 2021)
- REHEARSE-AF (Halcox et al., 2017)
- mSToPS (Steinhubl et al., 2018)

**Issue**

These studies used prolonged or technology-assisted rhythm monitoring strategies that differed substantially from conventional opportunistic or invitation-based screening.

**Assumption Applied**

These interventions were categorized as device-based screening strategies for descriptive comparison purposes.

**Rationale**

This classification was used to facilitate interpretation of screening intensity and monitoring duration across studies.

**Assumption 3: Primary Care Applicability**

**Studies affected**

- STROKESTOP
- LOOP
- mSToPS

**Issue**

Some studies were conducted in community-based or population-based settings rather than routine primary care clinics.

**Assumption Applied**

These studies were retained because they evaluated AF screening strategies in populations relevant to primary care implementation and prevention.

**Rationale**

The objective of the review was to evaluate AF screening strategies applicable to older adults in community and primary care contexts.

**Assumption 4: Quantitative Synthesis Eligibility**

**Studies affected**

Included in quantitative synthesis:

- SAFE (Hobbs 2005)
- SAFE Follow-up Analysis (Fitzmaurice 2007)
- STROKESTOP
- LOOP
- SCREEN-AF

Excluded from quantitative synthesis:

- REHEARSE-AF
- Uittenbogaart
- VITAL-AF
- mSToPS

**Issue**

Not all studies reported AF detection outcomes in directly comparable formats.

Assumption Applied

Studies were included in the structured quantitative synthesis only when sufficient numerical data and appropriate comparison groups were available.

**Rationale**

This approach avoided inappropriate pooling of heterogeneous outcome measures and screening methodologies.

**Statement Regarding Missing Data**

No study outcome values were estimated.

No event counts were reconstructed.

No missing numerical data were imputed.

No statistical assumptions were used to generate unreported outcomes.

Only descriptive classifications necessary for synthesis and interpretation were applied.
